# Epigenetic mechanisms in migraine: a promising avenue?

**DOI:** 10.1186/1741-7015-11-26

**Published:** 2013-02-04

**Authors:** Else Eising, Nicole A Datson, Arn MJM van den Maagdenberg, Michel D Ferrari

**Affiliations:** 1 Department of Human Genetics, Leiden University Medical Centre, Einthovenweg 20, Leiden, 2333 ZC, The Netherlands; 2 Department of Neurology, Leiden University Medical Centre, Albinusdreef 2, Leiden, 2333 ZA, The Netherlands

**Keywords:** DNA methylation, epigenetics, GWAS, histone modifications, inflammation, migraine, stress, valproate

## Abstract

Migraine is a disabling common brain disorder typically characterized by attacks of severe headache and associated with autonomic and neurological symptoms. Its etiology is far from resolved. This review will focus on evidence that epigenetic mechanisms play an important role in disease etiology. Epigenetics comprise both DNA methylation and post-translational modifications of the tails of histone proteins, affecting chromatin structure and gene expression. Besides playing a role in establishing cellular and developmental stage-specific regulation of gene expression, epigenetic processes are also important for programming lasting cellular responses to environmental signals. Epigenetic mechanisms may explain how non-genetic endogenous and exogenous factors such as female sex hormones, stress hormones and inflammation trigger may modulate attack frequency. Developing drugs that specifically target epigenetic mechanisms may open up exciting new avenues for the prophylactic treatment of migraine.

## Background

Migraine is a common, disabling brain disorder typically characterized by attacks of 4 to 72 h of severe headache and associated autonomic and neurological symptoms [1]. In 30% of patients attacks may be preceded by neurological aura symptoms, the likely consequence of a wave of neuronal and glial depolarization called cortical spreading depression (CSD) Activation of the trigeminovascular system is responsible for migraine pain. Migraine affects over 15% of the general population, with around 10% of migraine patients suffering from weekly attacks [2]. Current acute and prophylactic treatments are effective in less than half of the patients [2], indicating the need for more effective drugs. Identifying factors that predispose to migraine attacks is therefore crucial to provide specific molecular targets to design novel migraine drugs.

**Figure 1:**
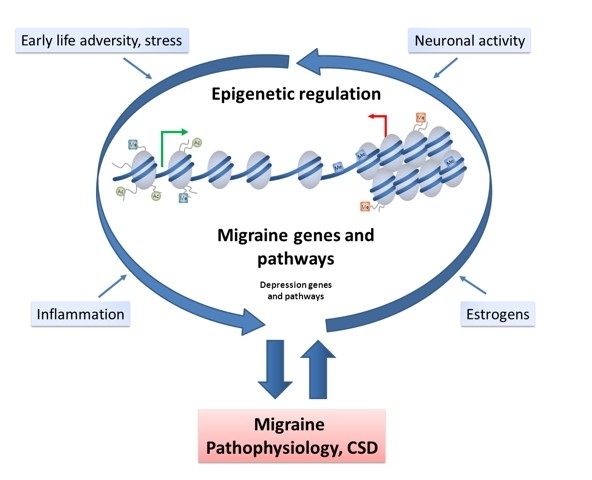
**Proposed model in which epigenetic factors influence migraine pathophysiology**. Different factors such as hormones, inflammation and neuronal activity can result in chromatin remodeling events affecting the expression of genes and pathways involved in the onset and progress of migraine and comorbid disorders such as depression. The hyperexcitability observed in migraine itself acts on the epigenome, thus creating a feed-forward loop resulting in chronification of migraine.

It is becoming increasingly clear that epigenetic processes play an important role in a wide variety of multifactorial diseases [3]. The question we aim to address here is: do they also play a role in migraine? Epigenetics encompasses changes to the DNA structure without changing the genetic code, resulting in chromatin remodeling and consequently affecting transcriptional potential and expression of genes. The main epigenetic modifications or 'marks' are post-translational modifications of the tails of histone proteins and DNA methylation, collectively comprising the epigenome (Figure 1). These marks are catalyzed by enzymes including histone deacetylases (HDACs), histone acetyltransferases (HATs) and DNA methyltransferases (DNMTs). Epigenetic marks can be dynamic but can also be stably inherited through cell divisions. Therefore, epigenetic processes enable cell and developmental stage-specific regulation of gene expression, but also play an important role in programming lasting responses to environmental cues.

This review highlights current evidence for an epigenetic link with migraine and speculates on neurobiological pathways that may be programmed by epigenetic mechanisms.

### Environmental factors and migraine

Although there is a clear genetic component to migraine, environmental factors likely contribute approximately equally to the risk of developing migraine [4]. Environmental factors may directly trigger migraine attacks or lower the attack threshold by rendering the brain more susceptible to trigger factors. Modulators of migraine attack frequency include female sex hormones, given that migraine affects two to three times more females than males, and the occurrence is influenced by the menstrual cycle and pregnancy, as well as by hormonal contraceptives [5]. Moreover, menopause, with its reduced estrogen and progesterone production, is associated with a decline in migraine attack frequency [6].

Animal studies have provided further evidence that female hormones can affect mechanisms directly involved in migraine pathophysiology. For instance, female transgenic migraine mice carrying a pathogenic gene mutation that causes familial hemiplegic migraine (FHM) in humans [7] have an increased susceptibility for CSD induction compared to male transgenic migraine mice [8,9]. Ovariectomy of these female migraine mice reduced the susceptibility for CSD induction, which was partially abrogated by estrogen replacement [9]. In addition, several studies in rats showed that estrogen treatment, ovariectomy as well as the menstrual cycle can alter the activity of the trigeminal nociceptive pathway [10]. The effects of female hormones are predominantly transmitted via nuclear receptors that adjust epigenetic programming of their target genes [11]. For instance, estrogen receptor β regulates expression of glucose transporter *Glut4 *by maintaining a low level of DNA methylation at its promoter, thereby enabling its activation [12]. Notably, treatment of mice with an estrogen receptor β agonist increased γ-aminobutyric acid (GABA) synthesis [13], whereas estrogen receptor α activation enhanced expression of astrocytic glutamate transporter *Glast *(also known as *Slc1a3 *and *Eaat1*) [14], thereby changing the balance between inhibitory and excitatory neurotransmission. This balance seems to be affected in migraine, resulting in increased excitatory neuronal activity, that is, creating hyperexcitability [15]. This is supported by the observation in a transgenic FHM1 mouse model that excitatory, glutamatergic, cortical neuronal activity was increased [16].

Another environmental factor that can modulate the occurrence of migraine attacks is stress. The stress system is highly sensitive to environmental programming through epigenetic mechanisms. The first study that assessed the effect of stress on the epigenome showed that low maternal care, a model for early life stress, could lastingly affect the behavior and stress responsiveness of rat offspring throughout their lifespan through increased DNA methylation at the brain-specific promoter of the glucocorticoid receptor gene *Nr4a3*, the main receptor for glucocorticoid stress hormones [17]. Other studies have linked stress during early life as well as during adulthood to a wide variety of long-lasting epigenetic alterations at stress effector genes (for example, *Bdnf*, *Gr *and *Crh*), affecting structural and functional aspects of the brain such as stress reactivity and synaptic plasticity [18]. In migraine, short-lasting stressful periods are among the most frequently reported trigger factors [19]. Moreover, early life stress may lead to an increased risk for migraine in humans [20] and severe acute stress during adult life may promote migraine as well, given the evidence that post-traumatic-stress disorder seems to be increased in migraine patients [21]. Interestingly, early life stress is a risk factor for developing depression [22], a disorder with increased comorbidity with migraine (see below), and first evidence has also associated it with alterations of the immune system [23,24], a condition that is also associated with migraine (see below). It is therefore conceivable that stress may produce long-lasting changes in the threshold for migraine attacks by inducing epigenetic modifications throughout the brain.

### Epigenetics in comorbidities of migraine

Depression and epilepsy are two disorders that display bidirectional comorbidity with migraine. Moreover, migraine is associated with an increased risk of cardiovascular disease including stroke and myocardial infarction [25]. Interestingly, depression also shares modulatory factors with migraine, such as female hormones and chronic stress, the latter of which is an established risk factor for depression [26]. A role for epigenetics has been suggested for all these comorbid disorders of migraine, and has already been extensively reviewed [27-29]. In summary, the main proof for a role of epigenetic mechanisms in depression is evident from animal models for major depressive disorder that show large changes in epigenetic programming of stress related genes (for example, *Bdnf*) that could be reversed by antidepressant treatment [30,31]. Moreover, a recent study reported differential expression of DNMTs in peripheral white blood cells of patients with major depressive disorder and bipolar disorder, suggesting that aberrant epigenetic gene regulation may be associated with the pathophysiology of mood disorders [32]. The contribution of epigenetics in epilepsy is illustrated by the high occurrence of this disorder in Rett syndrome and alpha thalassemia mental retardation, two disorders caused by mutations in the epigenetic effector proteins methyl CpG binding protein 2 (MeCP2) and ATRX, respectively [33,34]. In addition, in the brain of temporal lobe epilepsy patients increased DNA methylation was found at the promoter of Reelin [35], a gene involved in brain plasticity whose reduced expression contributes to epilepsy pathogenesis [36]. Also in blood and tissue of cardiovascular disease patients, as well as in cardiovascular disease models, aberrant DNA methylation levels were found, both globally and at cardiovascular disease associated genes [27]. Therefore, causal pathways shared between migraine and its comorbid disorders may be modulated by epigenetic mechanisms.

### Epigenetics and chronification of migraine

Attack frequency may change over the lifetime of a migraine patient and in some patients develops into chronic migraine with attacks more than 15 days per month [2]. As migraine patients with a high baseline attack frequency have an increased risk for developing chronic migraine [37], migraine attacks themselves might promote the development of chronic migraine. Additionally, recent studies have shown that synchronous neuronal activity, such as occurs during CSD, results in changes in epigenetic marks at genes involved in neuronal plasticity and neuroprotection [38-40]. This fits with evidence that epigenetic mechanisms are involved in the regulation of basal synaptic activity (that is, long-term changes in synaptic activity levels) [41]. It is, therefore, conceivable that increased neuronal activity in migraine alters the brain epigenome, thereby promoting the subsequent migraine attacks and creating a feed-forward loop (Figure 1) in which epigenetic programming of genes and pathways underlying excitability are altered towards a more sensitive baseline.

### Inflammation, migraine and epigenetics

Inflammation has long been suggested to play a role in migraine. Firstly, proinflammatory cytokines are released during CSD in rat hippocampus [42]. Interestingly, inflammatory mediators can induce gene expression changes by altering epigenetic marks [43], while their expression can be inhibited by HDAC inhibitor treatment [44]. Secondly, immune mediators are involved in sensitization of nerve endings in the meninges that promote the feeling of pain [45]. This sensitization effect is the result of vasodilation and the release of proinflammatory cytokines. Prolonged inflammatory pain was shown to promote pain sensitivity by causing histone hypoacetylation at the *Gad2 *gene, which is involved in GABAergic signaling [46]. Therefore, migraine-related pain may cause sensitization of certain pain pathways via inflammation-induced changes in epigenetic gene regulation.

### Epigenetic risk factors for migraine

Many candidate gene association studies and recently also genome-wide association studies (GWAS) have searched for genetic factors underlying migraine heritability. Some genetic factors increasing migraine susceptibility have direct links to epigenetic mechanisms. For instance, DNA polymorphisms in *MTHFR *(the gene for 5',10'-methylenetetrahydrofolate reductase), a gene required for DNA methylation, show a slight association with migraine in some studies [47]. More recently, GWAS have identified several single nucleotide polymorphisms linked to migraine pathophysiology [48-50] in genes or in regulatory regions of genes involved in epigenetic processes, including *MTDH*, *MEF2D *and *PRDM16*. For example, metadherin (MTDH) associates with nuclear factor κB (NFκB) and a HAT to promote the expression of NFκB target genes [51]. Myocyte enhancer factor 2D (MEF2D) can target methyltransferase complexes to specific genes to mark them for gene expression [52]. *MEF2 *has recently been shown to be regulated via the glucocorticoid receptor [53], which may be one of the mechanisms by which stress hormones affect the epigenome. Finally, PR domain containing 16 (PRDM16) is involved in positioning and removing specific chromatin modifications at enhancer regions of Notch target genes during olfactory neuron differentiation in drosophila [54]. These studies suggest that some of the migraine GWAS hits may contribute to developing migraine through epigenetic modifications at their target genes. Despite great efforts, GWAS have until now only explained a fraction of the total heritability of migraine. One explanation for this so-called 'missing heritability' is the fact that GWA approaches are unsuited for capturing disease susceptibility DNA variants with a low allele frequency, but which are expected to have a larger effect size [55]; next generation sequencing is currently used to identify such variants. Another possible explanation could be that DNA is not the only carrier of heritable information; epigenetic information can also be transmitted across cell divisions and possibly even transgenerationally. Recent techniques that couple array-based analysis or next generation sequencing to methods to study epigenetic marks enable genome-wide and high-throughput analysis of epigenetic marks. These techniques can analyze histone modifications (that is, by chromatin immunoprecipitation (ChIP)) as well as DNA methylation (that is, by bisulfite conversion of unmethylated cytosines or by immunoprecipitation of methylated DNA using antibodies (MeDIP) or methyl binding domains (MBD)) [56]. It therefore seems likely that the recently proposed epigenome-wide association studies, that can associate epigenetic marks to a trait (in addition to genetic variations found by GWAS) [57], will soon be put to use to further discover factors involved in migraine heritability. Because brain tissue from migraine patients is scarcely available, it may be feasible to use DNA banks consisting of large collections of stored DNA samples of migraine sufferers as a resource for identification of heritable DNA methylation marks that predispose to migraine.

### Potential for migraine drugs with epigenetic action

As epigenetic regulation of gene expression is a dynamic and reversible process, it is a perfect target for drugs. Interestingly, one of the current prophylactic drugs for migraine (valproate) is a HDAC inhibitor that facilitates chromatin remodeling. Valproate is an anticonvulsive drug with many modes of action and side effects [58]; therefore, it is not certain that its beneficial effects result from its effects on the epigenome. Interestingly, valproate is also successfully used in the treatment of bipolar depression [59]. Furthermore, in animal models for depression, reduced HDAC activity is required for the restoration of the effects of chronic stress on the epigenome by antidepressant treatment [30].

While several epigenetic drugs are in clinical trials for the treatment of cancer, to date (with the exception of valproate) there are currently no epigenetic drugs that have gone into clinical trials for the treatment of neuropsychiatric disorders or migraine [60]. Developing drugs that specifically target epigenetic mechanisms in the brain will open up exciting new avenues for the prophylactic treatment of migraine.

## Conclusions

Migraine is a common and disabling brain disease. Today its etiology is only partially known. In a subgroup of patients the migraine attack frequencies may dramatically increase up to near daily attacks, affecting their daily life, but the exact mechanism for chronification is unknown. Epigenetic mechanisms may underlie a part of migraine pathophysiology (and even the chronification of migraine) and therefore might provide a novel promising avenue for improving pharmacotherapy. More research is required to identify (epigenetic) targets that affect migraine pathophysiology as well as epigenetic drugs that specifically act to modulate chromatin structure at migraine pathways and can be used as a target in the prophylactic treatment for migraine.

## Abbreviations

ChIP: chromatin immunoprecipitation; CSD: cortical spreading depression; DNMTs: DNA methyltransferases; GWAS: genome-wide association study; HATs: histone acetyltransferases; HDACs: histone deacetylases; MBD: methyl binding domains; MeDIP: methylated DNA immunoprecipitation.

## Competing interests

The authors declare that they have no competing interests.

## Authors' contributions

EE carried out the literature search and drafted the manuscript. NAD conceived the concept and design of the commentary, helped to draft the manuscript and revised it critically. AMJMvdM and MDF read and critically revised the manuscript. MDF initiated development of this commentary. All authors read and approved the final manuscript.

## Pre-publication history

The pre-publication history for this paper can be accessed here:

http://www.biomedcentral.com/1741-7015/11/26/prepub
